# G-CSF Inhibits Pulmonary Fibrosis by Promoting BMSC Homing to the Lungs via SDF-1/CXCR4 Chemotaxis

**DOI:** 10.1038/s41598-020-65580-2

**Published:** 2020-06-29

**Authors:** Fei-yan Zhao, Tian-yin Cheng, Lei Yang, Yan-hong Huang, Chen Li, Jian-zhong Han, Xiao-hong Li, Li-juan Fang, Dan-dan Feng, Yi-ting Tang, Shao-jie Yue, Si-yuan Tang, Zi-qiang Luo, Wei Liu

**Affiliations:** 1 Department of Physiology, Xiangya School of Medicine, Central South University, Changsha, Hunan 410008 China; 2 College of Veterinary Medicine, Hunan Agricultural University, Changsha, Hunan 410128 China; 3 Xiangya Nursing School, Central South University, Changsha, Hunan 410013 China; 4 Department of Physiology, Changzhi Medical College, Changzhi, Shanxi 046000 China; 5 Department of Pediatrics, Xiangya Hospital, Central South University, Changsha, Hunan 410008 China

**Keywords:** Respiration, Mesenchymal stem cells

## Abstract

Bone marrow mesenchymal stem cells (BMSCs) have multi-lineage differentiation potential and play an important role in tissue repair. Studies have shown that BMSCs gather at the injured tissue site after granulocyte-colony stimulating factor (G-CSF) administration. In this study, we first investigated whether G-CSF could promote BMSC homing to damaged lung tissue induced by bleomycin (BLM) and then investigated whether SDF-1/CXCR4 chemotaxis might be involved in this process. Next, we further studied the potential inhibitory effect of G-CSF administration in mice with lung fibrosis induced by bleomycin. We examined both the antifibrotic effects of G-CSF in mice with bleomycin-induced pulmonary fibrosis *in vivo* and its effects on the proliferation, differentiation and chemotactic movement of cells *in vitro*. Flow cytometry, real-time PCR, transwell and Cell Counting Kit-8 (CCK-8) assays were used in this study. The results showed that both preventative and therapeutic G-CSF administration could significantly inhibit bleomycin-induced pulmonary fibrosis. G-CSF enhanced BMSC migration to lung tissues, but this effect could be alleviated by AMD3100, which blocked the SDF-1/CXCR4 axis. We also found that BMSCs could inhibit fibroblast proliferation and transdifferentiation into myofibroblasts through paracrine actions. In conclusion, G-CSF exerted antifibrotic effects in bleomycin-induced lung fibrosis, in part by promoting BMSC homing to injured lung tissues via SDF-1/CXCR4 chemotaxis.

## Introduction

Idiopathic pulmonary fibrosis (IPF) is a progressive interstitial lung disease characterized by extensive proliferation of fibroblast (FB) and deposition of extracellular matrix resulting in the formation of fibroblastic foci, the signature morphological lesion of IPF^1^. Currently, there are no specific and effective treatment methods, and the mortality rate of IPF is extremely high^2^. Therefore, searching for an effective treatment is urgently needed.

With the rapid development of stem cell biology, stem cell therapy has become a popular area of research in the medical field. Mesenchymal stem cells (MSCs) are multipotent stem cells, and their highly plastic and differentiation abilities hold extensive promise for clinical application^3^. Researchers have investigated that MSCs play a vital role in tissue repair by paracrine signals and directly differenting into substitute functional cells^4^. Mesenchymal stem cells have been applied to the study of clinical diseases such as the nervous system, cardiovascular system and respiratory system^5–8^. However, the administration of exogenous MSCs is limited due to various factors, including complex production techniques and high costs. Therefore, developing effective methods for promoting autologous MSC participation in tissue repair has become an alternative approach in stem cell research and is of great significance for improving clinical treatments.

BMSCs are an important source of MSCs *in vivo*, and they play an important role in tissue repair. G-CSF is a strong bone marrow stem cell mobilizer. Several studies have shown that BMSCs, but not haemopoietic stem cells (HSCs), mobilized by G-CSF promote the repair of various damaged tissue types^9,10^. Although its potent of mobilizing bone marrow stem cells is known, whether G-CSF participates in tissue repair via other mechanisms has not yet been fully elucidated.

Stromal cell-derived factor-1 (SDF-1), a chemokine of the CXC family. Studies have found that SDF-1 and its specific receptor CXCR4 play an important role in stem cell mobilization, chemotaxis, homing and colonization of damaged myocardial tissue^11,12^. In locally damaged tissue, SDF-1 expression is upregulated, which causes the BMSCs to migrate along the concentration gradient of SDF-1 via their surface receptor CXCR4 to the injured tissue^13,14^. Furthermore, that up-regulation of the expression of CXCR4 on the surface of BMSCs also promote more BMSCs to migrate to damaged tissues, and will be more conducive to tissue damage repair.

At present, the potential therapeutic effect of G-CSF mobilizes autologous BMSCs in the treatment of pulmonary fibrosis remains unclear. The aim of our study was to investigate whether mobilization of autologous BMSCs by G-CSF could inhibit pulmonary fibrosis, and further explore the mechanism of action. Our results provided the first evidence that G-CSF could promote the migration of BMSCs to damaged lung tissue through upregulating the expression of CXCR4 on BMSCs, which could effectively alleviate pulmonary fibrosis.

## Results

### G-CSF inhibited bleomycin induced lung fibrosis

**Figure 1:**
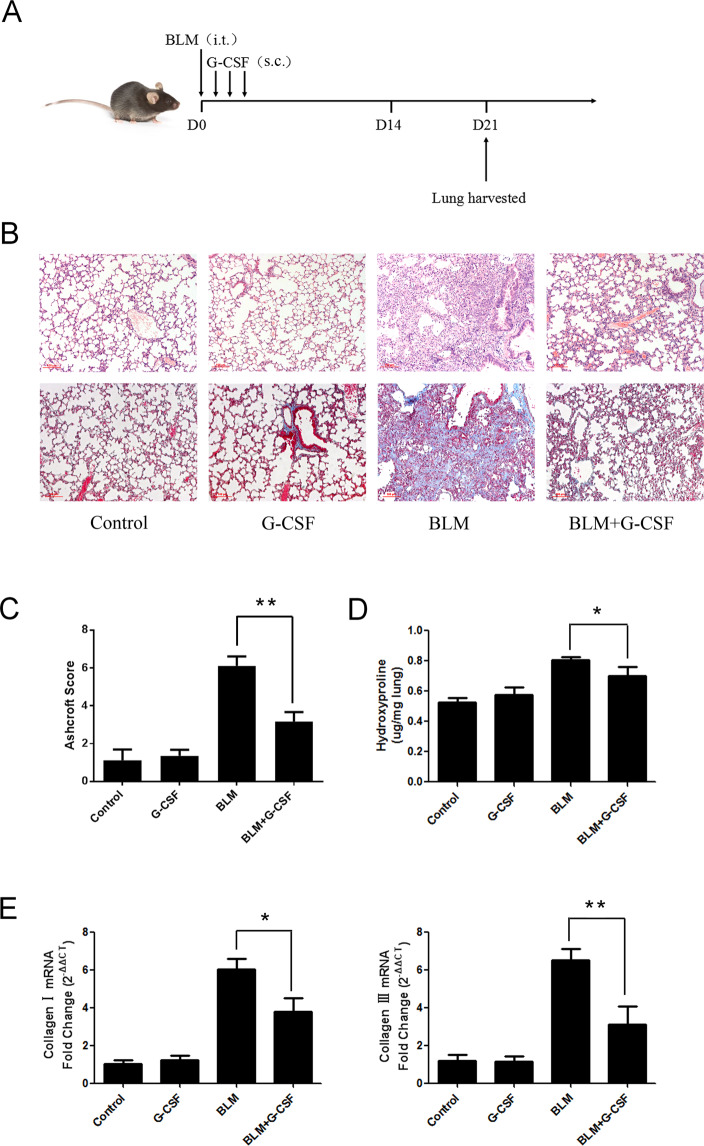
The protective effects of preventative G-CSF treatment on BLM-induced pulmonary fibrosis. (**A**) Experimental design 1: Preventative treatment with G-CSF (40 µg/kg) was performed daily from day 1 to day 3. Mice were sacrificed, and lung tissues were harvested on day 21 for further testing. (**B**) The pathological examination of the lung sections utilized H&E staining (upper row) and Masson’s trichrome staining (lower row) (scale bar =100 µm, ×100). (**C**) The Ashcroft scoring method was used to determine the severity of pulmonary fibrosis. (**D**) The collagen content was quantified by HYP assay. (**E**) The mRNA expression levels of collagen I and III were also determined by real-time PCR. n = 4–6. *P < 0.05, **P < 0.01 between the indicated groups. Bars: mean ± SD.

**Figure 2:**
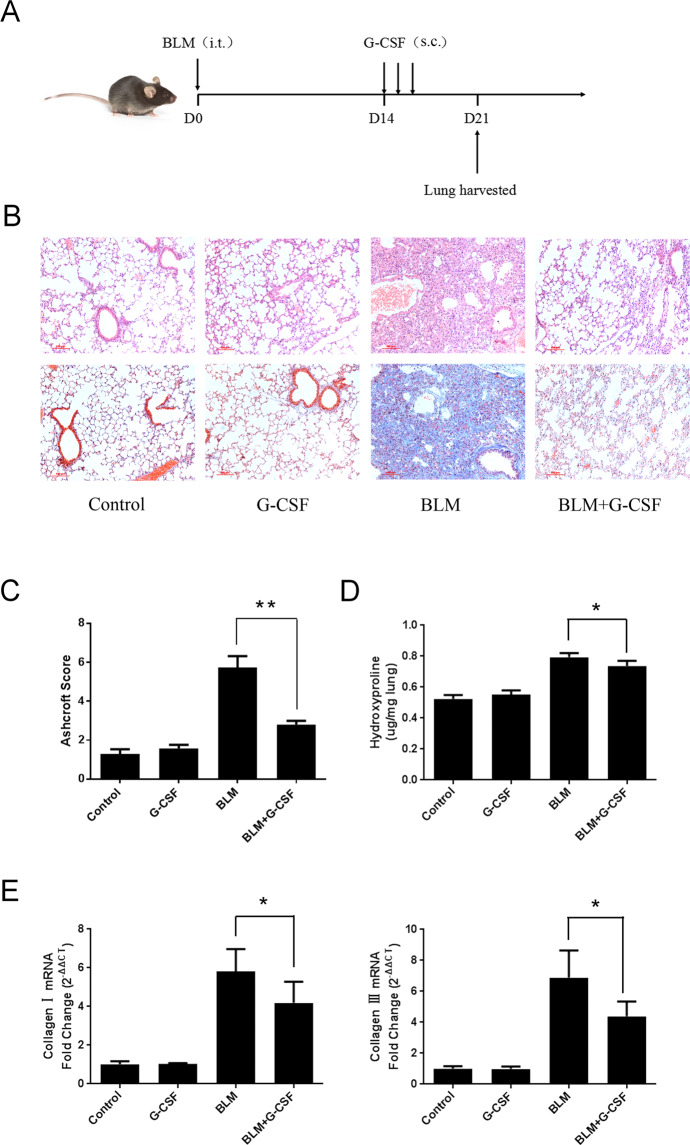
The protective effects of therapeutic G-CSF treatment on BLM-induced pulmonary fibrosis. (**A**) Experimental design 2: therapeutic treatment with G-CSF (40 µg/kg) was performed daily from day 14 to day 16. Mice were sacrificed, and lung tissues were harvested on day 21 for further analysis. The degree of pulmonary fibrosis was estimated by (**B**) histopathological examination, (**C**) histopathological score, (**D**) HYP assay, and (**E**) collagen I and III mRNA expression. n = 3–5. *P < 0.05, **P < 0.01 between the indicated groups. Bars: mean ± SD.

Female C57BL/6 mice were injected intratracheally with BLM (5 mg/kg) on day 0. Two schedules were designed to better evaluate the effects on pulmonary fibrosis. For preventative treatment, the mice were treated with G-CSF (40 and 60 µg/kg) for 3 consecutive days starting on day 1 after BLM was injected (Fig. 1A). The obvious fibrosis foci was observed in bleomycin treated mice by pathological examination of the lung sections utilizing H&E staining and Masson’s trichrome staining. In contrast, lung fibrosis was obviously alleviated in the G-CSF (40 µg/kg)-treated groups (Fig. 1B,C). Collagen I and III mRNA expression in the BLM-induced model remarkably increased, as well as the HYP content. However, G-CSF (40 µg/kg) significantly reduced these three indicators (Fig. 1D,E). When the mice were treated with G-CSF at a dose of 60 µg/kg, no significant inhibitory effect on BLM-induced lung fibrosis was observed in the treatment groups (Supplementary figure 1). For therapeutic treatment, G-CSF was injected subcutaneously for 3 consecutive days starting on day 14 after BLM was injected intratracheally. Consistent with the preventative treatment results, the therapeutic administration of G-CSF (40 µg/kg) also alleviated pulmonary fibrosis (Fig. 2). Our results show that G-CSF exerts antifibrotic effects in bleomycin-induced lung fibrosis.

### G-CSF increased the number of MSCs in BLM-treated lungs

**Figure 3:**
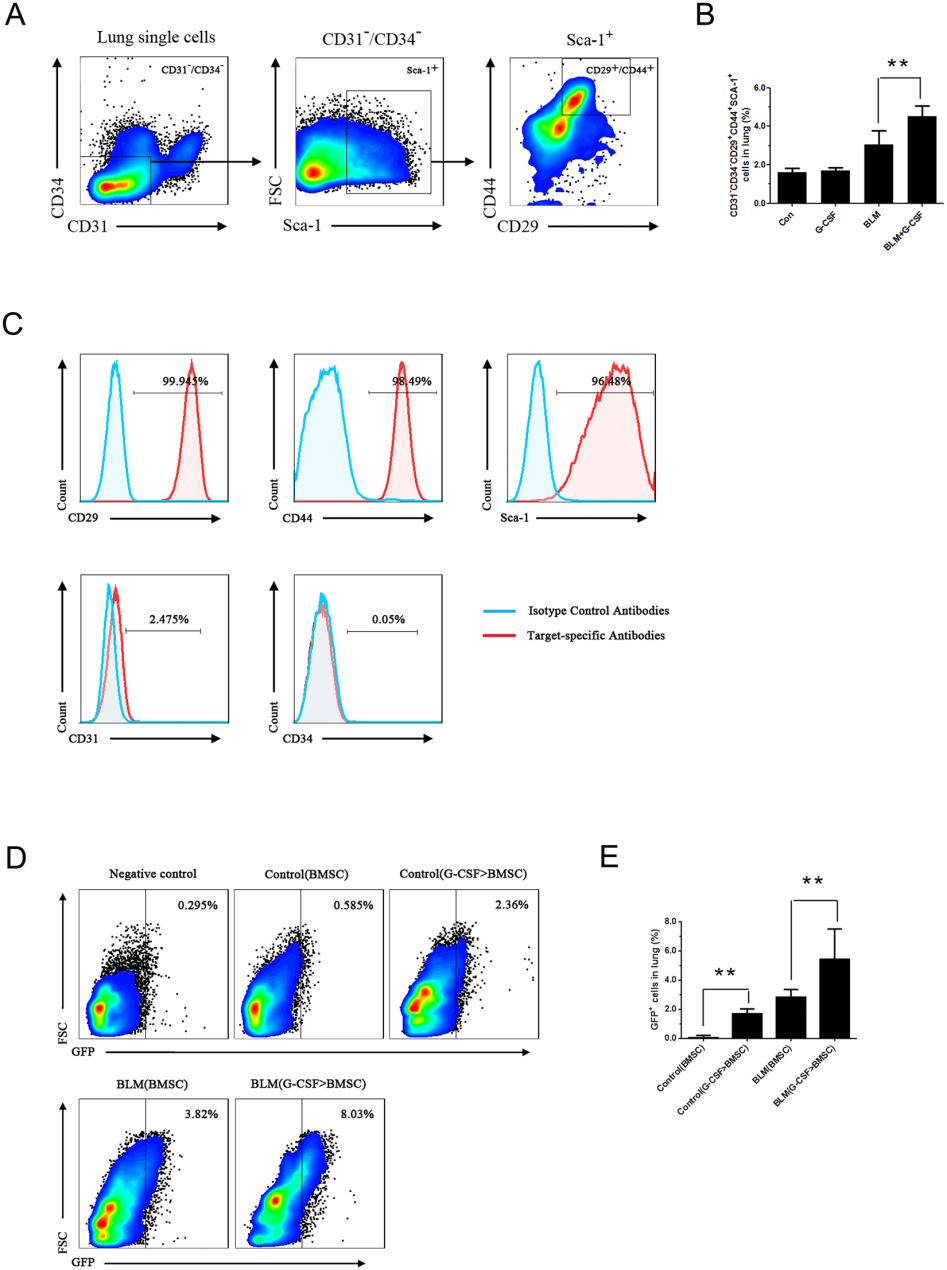
G-CSF increased the number of MSCs in BLM-treated lungs and promoted BMSC homing to lung tissues. (**A**) FCM gating strategy for the surface expression of CD31^-^CD34^-^CD29^+^CD44^+^Sca-1^+^ cells from mouse lungs. (**B**) The number of CD31^-^CD34^-^CD29^+^CD44^+^Sca-1^+^ cells in the lungs was analysed by FCM. (**C**) Identification of BMSCs in mice by FCM. Representative flow cytometric analysis of BMSCs isolated from the bone marrow and stained with antibodies against CD29, CD31, CD34, CD44 and Sca-1. (**D**) Representative FCM analysis of the GFP-labelled BMSC population. GFP-labelled BMSCs were infused into mice, and after 36 hours, the lungs were prepared into a single-cell suspension and analysed by FCM. (**E**) Data analysis of the GFP-labelled BMSC population tested by FCM. Control (BMSC) and BLM (BMSC) represent conditions where BMSCs were not pretreated before being infused into control or BLM mice, whereas control (G-CSF > BMSC) and BLM (G-CSF > BMSC) represent conditions where BMSCs were pretreated with G-CSF (30 ng/mL) before being infused into control or BLM mice. n = 3–5. *P < 0.05, **P < 0.01 between the indicated groups. Bars: mean ± SD.

To identify the mechanism underlying the antifibrotic effect of G-CSF, we used flow cytometry to measure the number of MSCs in lung tissue (Fig. 3A). The data display the number of MSCs was significantly higher in G-CSF-treated lungs than in BLM-treated lungs (Fig. 3B). These results suggested that G-CSF increases the number of MSCs in BLM-treated lungs.

### G-CSF preconditioning enhanced the homing activities of BMSCs after their infusion into mice

BMSCs are an important component of MSCs *in vivo* and play an important role in tissue repair. To determine whether an incline of MSCs in the lung is associated with an increase in BMSCs, we first observed the direct effect of G-CSF on the homing activities of BMSCs. BMSCs were phenotypically identified by a lack of expression of the immune markers CD31 and CD34 and positive expression of CD29, CD44 and Sca-1 on the cell surface, as determined by FCM (Fig. 3C). Exogenous GFP-labelled BMSCs with or without G-CSF (30 ng/mL) preconditioning were infused into the tail veins of the mice. Thirty-six hours after infusion, the number of GFP-labelled BMSCs in the lung tissue was counted by FCM. In both control and BLM-treated mice, the G-CSF pretreated BMSCs exhibited better homing efficiency than the nontreated BMSCs (Fig. 3D,E). Therefore, our results indicated that G-CSF promoted BMSC homing to lung tissues.

### G-CSF preconditioning enhanced the migration of BMSCs via SDF-1/CXCR4 chemotaxis

**Figure 4:**
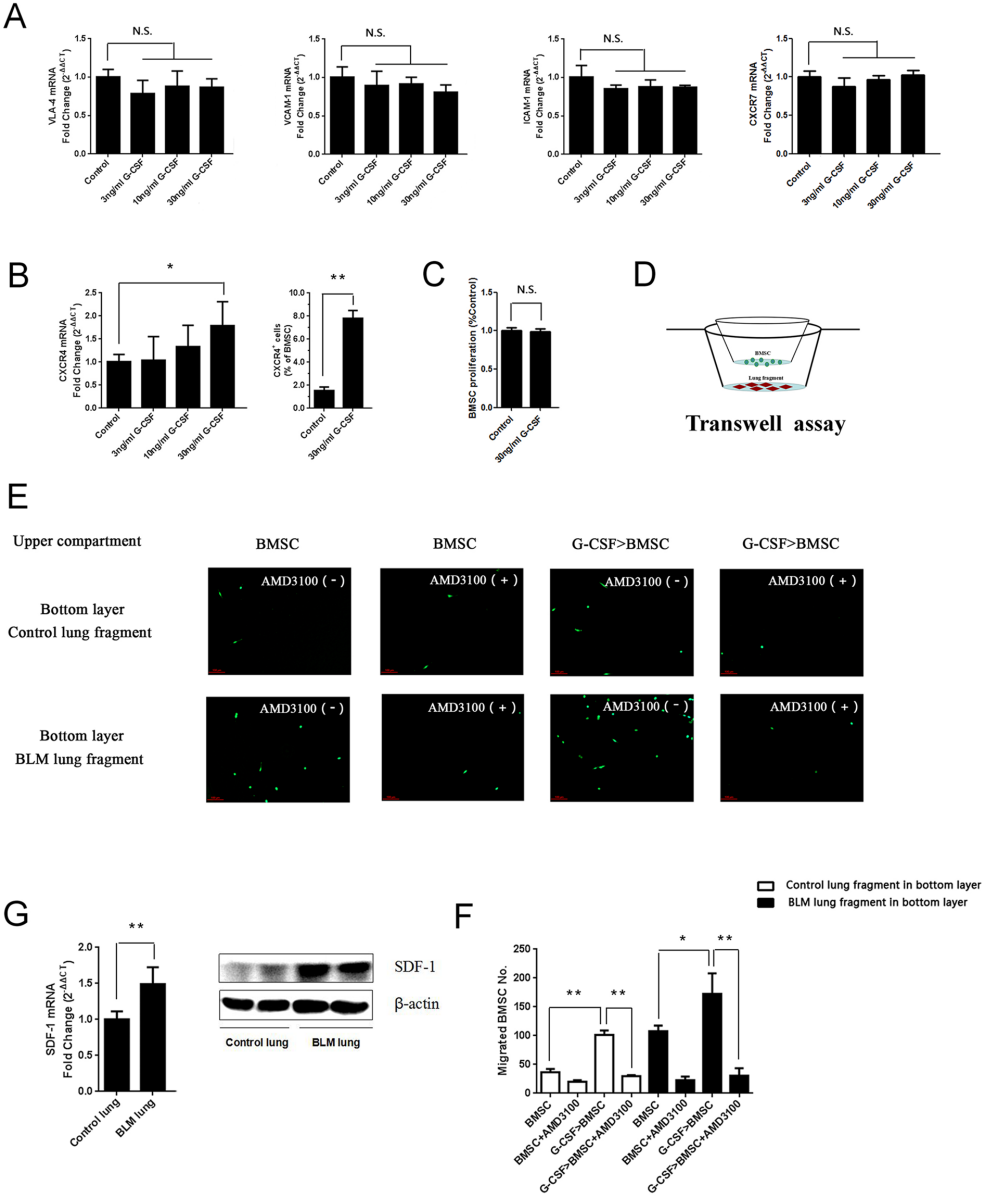
G-CSF preconditioning promoted BMSC migration via the SDF-1/CXCR4 axis *in vitro*. (**A**) The expression levels of VLA-4, ICAM-1, VCAM-1 and CXCR7 were quantified by real-time PCR. (**B**) The expression of CXCR4 was quantified by real-time PCR and FCM at 24 hours after G-CSF treatment. (**C**) The proliferation of BMSCs with or without G-CSF (30 ng/mL) was tested by CCK-8 assay. (**D**) Experimental design of the transwell assay: lungs collected from control and BLM mice were cut into fragments and placed in the lower transwell chamber. In the upper compartment of the transwell chamber, GFP-labelled BMSCs with or without G-CSF (30 ng/mL) pretreatment were seeded to test chemoattraction. (**E**) Representative image of migrated GFP-labelled BMSCs (green). (**F**) Analysis of GFP-labelled BMSC migration. (**G**) The mRNA and protein expression of SDF-1 in control and BLM-treated lungs as quantified by real-time PCR and Western blotting. n = 3. *P < 0.05, **P < 0.01 between the indicated groups. Bars: mean ± SD.

To determine how G-CSF increases BMSC homing efficiency, mRNA expression levels of the representetive molecules of adhesion Vascular cell adhesion molecule-1 (VCAM-1), Intercellular cell adhesion molecule-1 (ICAM-1), and Very late antigen-4 (VLA-4) as well as the CXC chemokine receptors-7 (CXCR7) and CXCR4 on the surface of the BMSCs were investigated. The results showed that G-CSF treatment affected neither the expression of the adhesion molecules mentioned above nor the expression of CXCR7. However, G-CSF treatment obviously increased the mRNA and protein level of CXCR4, which plays vital roles in the chemotactic activity of various cell types (Fig. 4A,B). In our study, G-CSF showed no effect on promoting BMSC proliferation (Fig. 4C). To evaluate whether BMSCs were recruited via the SDF-1/CXCR4 axis, a transwell assay was conducted. We seeded nontreated or G-CSF (30 ng/mL)-pretreated BMSCs into the upper transwell compartment with or without AMD3100, a CXCR4 antagonist, and control lungs or BLM-treated lungs were cut into small pieces and placed in the lower chamber (Fig. 4D). After 18 hours, the migrated GFP-labelled BMSCs from each group were counted. The results showed that BMSCs were obviously recruited by BLM-treated lung tissue in co-culture, and the G-CSF (30 ng/mL)-pretreated BMSCs migrated more than the nontreated BMSCs; however, these outcomes were inhibited when the SDF-1/CXCR4 axis was blocked by AMD3100 (Fig. 4E,F). SDF-1 in the BLM-treated lungs was also significantly upregulated compared with that in the control lungs (Fig. 4G). Collectively, these results revealed that G-CSF enhanced the migration of BMSCs in a manner dependent on SDF-1/CXCR4 chemotaxis.

### G-CSF preconditioning did no affect on the paracrine actions of BMSCs on inhibiting FB proliferation and transdifferentiation

**Figure 5:**
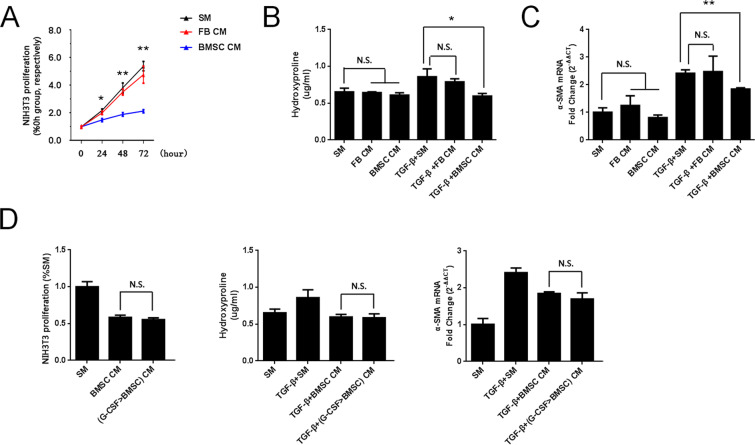
G-CSF preconditioning could not enhance the paracrine actions of BMSCs on inhibiting FB proliferation and transdifferentiation. (**A**) Cell proliferation was measured by CCK-8 assay after culture with different media. (**B**) HYP content in the supernatant and (**C**) α-SMA mRNA expression were quantified at 24 hours after various treatments. (**D**) Cell proliferation, HYP content and α-SMA mRNA expression were measured after culture with various media for 24 hours. SM: standard medium. FBCM: NIH3T3-conditioned medium. BMSC CM: BMSC-conditioned medium. G-CSF > BMSC CM: G-CSF > BMSC-conditioned medium. n = 3. *P < 0.05, **P < 0.01 represent BMSC CM vs SM in Figure 6A. Bars: mean ± SD.

In recent years, studies have shown that BMSC paracrine functions play critical roles in tissue repair. Therefore, in this study, the effects of BMSC-conditioned medium on the proliferation and transdifferentiation of FBs were investigated. The proliferation of NIH3T3 cells was significantly lower in BMSC-conditioned medium than in standard or FB-conditioned medium (Fig. 5A). Regarding HYP content and α-Smooth muscle actin (α-SMA) expression levels without TGF-β stimulation, there was no difference between the various groups. However, after inducing Transforming growth factor-β (TGF-β), these two indicators were lower when using BMSC-conditioned medium than when using standard or FB-conditioned medium (Fig. 5B,C). These results indicated that BMSCs attenuated the proliferation and transdifferentiation of FBs through paracrine actions. In addition, we further investigated whether G-CSF (30 ng/mL) increased the homing activity of BMSCs and enhanced the paracrine actions of BMSCs. Our data showed that G-CSF (30 ng/mL) pretreatment could not enhance the effect of the paracrine actions of BMSCs on inhibiting FB proliferation and transdifferentiation (Fig. 5D).

## Discussion

BLM-induced pulmonary fibrosis in mice has been widely used as a model to study the pathophysiological mechanisms of IPF. Typical morphologic features of lung fibrosis were observed on day 14 in BLM-induced pulmonary injury^15,16^. Therefore, two schedules were designed to better evaluate the treatment effects on pulmonary fibrosis. The results showed that both the preventative and therapeutic administration of G-CSF could significantly inhibit BLM-induced pulmonary fibrosis, fully confirming effects of G-CSF in antifibrosis.

Several studies have shown that BMSCs but not HSCs mobilized by G-CSF repair of many damaged tissue types^9,10^. Although its ability to mobilize bone marrow stem cells such as HSCs, BMSCs and endothelial progenitor cells (EPCs) is known, the mechanism of G-CSF involved in tissue repair remains unclear. Since HSCs can significantly aggravate lung injury, we focus on the therapeutic effects of BMSCs mobilized by G-CSF on pulmonary fibrosis, and the role of G-CSF-mobilized EPCs in improving pulmonary fibrosis will be further studied in subsequent experiments.

In this study, the effect of G-CSF on BMSCs homing to damaged tissues was investigated. Our data showed that the G-CSF treatment group has a significantly higher level of MSCs than the BLM group. BMSCs are an important source of MSCs *in vivo* and play an important role in tissue repair. To determine whether an increase in MSCs in the lungs is associated with an increase in BMSCs, we studied the direct effect of G-CSF on the homing activities of BMSCs. The results showed that more G-CSF-pretreated BMSCs than nontreated BMSCs entered the lung tissue. Therefore, we propose that G-CSF promoted BMSCs homing to the lung tissue.

Studies have shown that a variety of factors in damaged tissues could affect the migration of cells in the peripheral circulation, such as chemokines released from local injury sites, increased vascular permeability due to endothelial damage, and upregulated expression of vascular endothelial adhesion molecules^17–19^. In addition, cells in the blood circulation with increased surface adhesion molecule and chemokine receptor expression can also promote cell migration to damaged tissues^20–23^. In this study, expression of several major adhesion molecules, VCAM-1, ICAM-1, and VLA-4, and chemokine receptors, CXCR7 and CXCR4, was detected on the surface of BMSCs before or after G-CSF treatment. G-CSF treatment did not affect the expression of the adhesion molecules or CXCR7, but it did significantly upregulate the expression of CXCR4. Furthermore, G-CSF pretreatment could promote the chemotactic migration of BMSCs to lung tissues, as shown in Fig. 4E,F. However, after AMD3100 (a CXCR4 antagonist) intervention, this promotion effect was significantly inhibited. Thus, the SDF-1/CXCR4 axis might mediate the promotion effect of G-CSF on the chemotactic migration of BMSCs.

Few studies have been conducted regarding the effect of CXCR4 on bone marrow stem cells induced by G-CSF. Petit *et al*. found that G-CSF mobilized HSCs into the blood by directly downregulating the expression of SDF-1 in the bone marrow, and CXCR4 expression in HSCs was upregulated indirectly^24^. Because the expression of SDF-1 is obviously increased in injured tissue, this might be the mechanism underlying the rapid homing of HSCs to damaged tissue after entering the blood. Our research showed that G-CSF directly upregulated the expression of CXCR4 on BMSCs, and this might be an explanation for its promotion of BMSCs homing to damaged lung tissue by activating the SDF-1/CXCR4 axis.

BMSCs promote tissue repair by directly differentiating into substitute functional cells or by paracrine actions^25,26^. In recent years, studies have shown that BMSCs paracrine functions may play critical roles in tissue repair. In this study, the effects of BMSC-conditioned medium on the proliferation and transdifferentiation of FBs were investigated. The results showed that BMSC-conditioned medium could significantly inhibit the proliferation of FBs and prevent TGF-β-induced FBs from differentiating into myofibroblasts. In addition, we found that when G-CSF increased the homing activity of BMSCs, it could not enhance the effects of BMSCs on inhibiting the proliferation and transdifferentiation of FBs. Therefore, we indicated that G-CSF mainly increased the number of BMSCs in the lung by facilitating the migration of endogenous BMSC to lung damaged tissues, thus promoting the effect of endogenous BMSC on the tissue repair. This conclusion has also been supported in other part of our studies, in which we pretreated BMSCs with G-CSF *in vitro*, and then injected them into mice via tail vein. In that study, the results showed that pretreatment of BMSCs with G-CSF promotes the homing of exogenous BMSCs to the lung, leading to a marked increase in the antifibrotic effects of exogenous BMSCs^27^.

The effects of G-CSF on lung injury and fibrosis are uncontroversial^28,29^. Adachi K *et al*. (2003) showed that G-CSF (100 µg/kg/day) exacerbates the lung injury induced by intratracheal administration of bleomycin (20, 2,000 µg/200 µl/rat). The exacerbating effects of G-CSF seem to be associated not only with the marked infiltration of activated neutrophils but also with the severity of the underlying lung injury^28^. Zhang F *et al*. (2011) demonstrated that the mobilization of bone marrow-derived cells by G-CSF (50 µg/kg/day) has a protective effect against bleomycin-induced lung injury and fibrosis^29^. Based on these experimental results, we hypothesized that the effects of G-CSF on BLM-induced lung fibrosis may be related to the dose of G-CSF administered. In our results, we found that 40 µg/kg G-CSF had obvilously therapeutic effects on bleomycin-induced pulmonary fibrosis. However, when the dose of G-CSF was up to 60 µg/kg, no significant inhibitory effect on BLM-induced lung fibrosis was observed in the treatment groups (Supplementary figure 1), indicating that a relative low dose was critical for the antifibrotic effect of G-CSF. These results were speculated to be related to the following factors: G-CSF exerts biological effects on various bone marrow stem cell types, including BMSCs, HSCs and EPCs; thus, in addition to BMSCs, other types of bone marrow stem cells might also participate in the development of pulmonary fibrosis. For example, EPCs can differentiate into vascular endothelial cells and participate in repairing pulmonary fibrosis damage^30,31^. However, HSCs exacerbate the development of pulmonary fibrosis by paracrine action^32^. Therefore, it was hypothesized that different G-CSF doses and treatment durations may result in different types and quantities of bone marrow stem cells entering the lungs and that the overall effect would be a combination of the protective and pathogenic effects. However, our hypothesis requires further study.

In present study, we concluded that G-CSF exerted antifibrotic effects in bleomycin-induced lung fibrosis, in part by promoting BMSCs homing to injured lung tissues via SDF-1/CXCR4 chemotaxis. These findings provide insights into the therapeutic effect of G-CSF treatment in lung fibrosis and underline that BMSCs may act as an autologous cell resource for developing treatments of interstitial lung disease.

## Materials and Methods

### Ethics statement

The experiments were performed in accordance with the guidelines of the National Institutes of Health (NIH) and were approved by the Ethics Committee of Central South University (Changsha, China).

### Animal model and experimental design

Eighty of the female C57BL/6 mice were captured from JingDa Laboratory Animal Company (Changsha, China). After being anaesthetized by pentobarbital sodium, the mice were injected intratracheally with 50 μL of BLM (5 mg/kg) (Nippon kayaku, Japan) on day 0. To investigate the antifibrotic effects of G-CSF (Qilu-pharma, China), C57BL/6 mice were assigned randomly to one of the following groups: (1) the control group, intratracheal saline plus subcutaneous saline; (2) the G-CSF group, intratracheal saline plus subcutaneous G-CSF; (3) the BLM group, intratracheal BLM and subcutaneous saline; or (4) the BLM  +  G-CSF group, intratracheal BLM plus subcutaneous G-CSF. For preventative treatment, G-CSF (40 μg/kg/d) was administered subcutaneously from day 1 to day 3, and on day 21 lung tissues were harvested. For therapeutic treatment, G-CSF (40 μg/kg/d) was administered subcutaneously from day 14 to day 16, and lung tissues were harvested on day 21.

### Histopathology

Lung tissues were fixed with a 4% paraformaldehyde solution and then embedded in paraffin to prepare tissue sections for pathological examination. The sections were examined after staining with haematoxylin and eosin (H&E) and Masson’s trichrome. Two investigators blinded to group assignments analyzed the samples and determined the severity of fibrosis was assessed semiquantitatively according to the method described by Ashcroft^33^, with the average score of two investigators deemed the fibrosis score of each animal (with the average score of five fields deemed the fibrosis score of each animal).

### Hydroxyproline assay

The content of collagen was examined with a hydroxyproline (HYP) kit (Njjcbio, China) performed according to the manufacturer’s instructions.

### RT- PCR

Total RNA was extracted from lung tissues and BMSCs with RNAiso Plus (TaKaRa, Japan), and cDNA synthesis was performed using a First Strand cDNA Synthesis Kit. SYBR Green and a Bio-Rad CFX96 real-time PCR detection system were used for detection. The primer sequences for RT-PCR are shown in Table 1.

**Table 1:** Primer sequences for real-time PCR (forward and reverse).

Gene	Forward primer	Reverse primer
β-actin	GGCTGTATTCCCCTCCAT	CCAGTTGGTAACAATGCCATGT
Collagen I	GAGCGGAGAGTACTGGATCG	GCTTCTTTTCCTTGGGGTTC
Collagen III	GCTCCTCTTAGGGGCCACT	CCACGTCTCACCATTGGGG
α-SMA	TGGCTATTCAGGCTGTGCTGTC	CAATCTCACGCTCGGCAGTAGT
SDF-1	TGCCCTTCAGATTGTTGCAC	TTTTCCTTTTCTGGGCAGCC
CXCR4	GGAAACTGCTGGCTGAAAAG	CTGTCATCCCCCTGACTGAT
CXCR7	GCCATGTAACAGCAGCGACT	ATGCCGATCACGAAGATGAA
VCAM-1	CCCAAACAGAGGCAGAGTGT	TGAGCAGGTCAGGTTCACAG
VLA-4	TGTCTGTGTCCCTGTTTGGA	TTTGAGGGGCCTACAGAGAA
ICAM-1	AGATCACATTCACGGTGCTG	CTGGCCTCGGAGACATTAGA

### Acquisition of MSCs from lung tissue

The mice were anaesthetized, while the pulmonary circulatory system was perfused with PBS to flush the cells in the blood. Lung tissues were removed and minced mechanically. The methods for obtaining MSCs in lung tissue have been described in our previous study^27^.

### BMSC isolation and culture

GFP-labelled BMSCs were purchased from Cyagen Biosciences Incorporated (Guangzhou, China), and BMSCs were extracted from the femurs of young C57BL/6 mice (4 weeks). The methods for extraction, culture and purification of BMSCs have been described in our previous study^27^.

### Flow cytometry

Flow cytometry was used to identify the phenotypes of MSCs isolated from the bone marrow or lungs. The cultured BMSCs or single lung cells were incubated with FITC hamster anti-mouse CD29, BV421 rat anti-mouse CD31, Alexa Fluor^®^647 rat anti-mouse CD34, PE-Cy^TM^7 rat anti-mouse CD44 and PE rat anti-mouse Sca-1 (BD Bioscience, USA). The BMSCs were stained with an anti-CXCR4 antibody to detect CXCR4 cell surface expression. Antibodies of the corresponding isotype served as the isotype controls.

### BMSC homing assay

First, GFP-labelled BMSCs were treated or untreated with rmG-CSF (30 ng/mL) (Peprotech, USA) in DMEM/F12 medium supplemented with 5% FBS for 24 hours, and these cells were used as rmG-CSF-pretreated BMSCs and nontreated BMSCs, respectively. Then, the nontreated or rmG-CSF (30 ng/mL)-pretreated BMSCs (1 × 10^6^ in 100 μL) were injected into the tail veins of mice in control group or BLM group. At 36 hours after GFP-labelled BMSC infusion, the mice were sacrificed, and the lungs were harvested. BMSCs were quantified as the number of GFP-labelled cells by FCM.

### Transwell assay

First, GFP-labelled BMSCs were treated with or without rmG-CSF (30 ng/mL) in DMEM/F12 medium supplemented with 5% FBS for 24 hours, and these cells were used as rmG-CSF-pretreated BMSCs and nontreated BMSCs, respectively. Then, we seeded the nontreated BMSCs (1 × 10^4^ in 200 μL) or G-CSF (30 ng/mL)-pretreated BMSCs into the upper transwell compartment in 24-well plates with or without AMD3100 (100 µg/mL). The control lungs or BLM-treated lungs were cut into small pieces (1 mm^3^) and placed in the lower chamber. After migration of the GFP-labelled BMSCs for 18 h, and washed the non-migrated BMSCs on the upper side of the membrane were removed with a swab. The migrated GFP-labelled BMSCs were captured and counted.

### Preparation of BMSC- and FB-conditioned medium

BMSCs or NIH3T3 cells were cultured 24 hours in a basal medium containing no fetal bovine serum, and collected culture mediun and designated BMSC- or FB-conditioned medium. BMSCs were pretreated with G-CSF for 24 hours, and the cells were then washed and exposed to fresh culture medium. After an additional 24 hours, the culture medium was collected and designated G-CSF > BMSC-conditioned medium. The conditioned medium was filter-sterilized through a 0.22-µm filter and stored at −80 °C until use. Before use in experiments, the final concentration of FBS in the conditioned medium was adjusted to 5%. Fresh culture medium supplemented with 5% FBS was used as the standard medium.

### Cell proliferation assay

NIH3T3 cells (murine lung FBs) were acquired from the National Genetics Key Laboratory (Changsha, China). For the various treatment conditions, After plated in 96 well plates NIH3T3 cells were then cultured for 24–72 h. NIH3T3 cell proliferation was tested using a CCK-8 (Beyotime, China) according to the instructions.

### Statistical analysis

For two groups, the data were analyzed using Student’s t-test. For multiple groups, comparisons among multiple groups were analyzed with one-way ANOVA, followed by Tukey’s Multiple Comparison Test using GraphPad Prism 5.0. Data are shown as mean ± SD. Differences between groups with P values less than 0.05 were considered significant.

## Supplementary information

Supplementary Information.

## Data Availability

The datasets generated during and/or analysed during the current study are available from the corresponding author upon reasonable request.

## References

[1] Richeldi L, Collard HR, Jones MG (2017). Idiopathic pulmonary fibrosis. Lancet..

[2] Spagnolo P, Wuyts W (2017). Acute exacerbations of interstitial lung disease: lessons from idiopathic pulmonary fibrosis. Curr Opin Pulm Med..

[3] Wan H (2014). Update on therapeutic mechanism for bone marrow stromal cells in ischemic stroke. J Mol Neurosci..

[4] Spees JL, Lee RH, Gregory CA (2016). Mechanisms of mesenchymal stem/stromal cell function. Stem Cell Res Ther..

[5] Geiger S, Hirsch D, Hermann FG (2017). Cell therapy for lung disease. Eur Respir Rev..

[6] Hsuan YC, Lin CH, Chang CP, Lin MT (2016). Mesenchymal stem cell-based treatments for stroke, neural trauma, and heat stroke. Brain Behav..

[7] Gu W, Hong X, Potter C, Qu A, Xu Q. Mesenchymal stem cells and vascular regeneration. Microcirculation. **24**, (2017). 10.1111/micc.12324 27681821

[8] Eom YW, Kim G, Baik SK (2015). Mesenchymal stem cell therapy for cirrhosis: Present and future perspectives. World J Gastroenterol..

[9] Wu CC (2017). G-CSF-mobilized Bone Marrow Mesenchymal Stem Cells Replenish Neural Lineages in Alzheimer’s Disease Mice via CXCR4/SDF-1 Chemotaxis. Mol Neurobiol..

[10] Fukuda K, Fujita J (2005). Mesenchymal, but not hematopoietic, stem cells can be mobilized and differentiate into cardiomyocytes after myocardial infarction in mice. Kidney Int..

[11] Steingen C (2008). Characterization of key mechanisms in transmigration and invasion of mesenchymal stem cells. J Mol Cell Cardiol..

[12] Son BR (2006). Migration of bone marrow and cord blood mesenchymal stem cells *in vitro* is regulated by stromal-derived factor-1-CXCR4 and hepatocyte growth factor-c-met axes and involves matrix metalloproteinases. Stem Cells..

[13] Sugiyama T, Kohara H, Noda M, Nagasawa T (2006). Maintenance of the hematopoietic stem cell pool by CXCL12-CXCR4 chemokine signaling in bone marrow stromal cell niches. Immunity..

[14] Salemand HK, hiemermann C (2010). Mesenchymal stromal cells:current understanding and clinical status. Stem Cells..

[15] Liu W (2013). Antiflammin-1 attenuates bleomycin-induced pulmonary fibrosis in mice. Respir Res..

[16] Chaudhary NI, Schnapp A, Park JE (2006). Pharmacologic differentiation of inflammation and fibrosis in the rat bleomycin model. Am J Respir Crit Care Med..

[17] Xu X (2013). Stromal cell-derived factor-1 enhances wound healing through recruiting bone marrow-derived mesenchymal stem cells to the wound area and promoting neovascularization. Cells Tissues Organs..

[18] Yan M (2017). Endothelial cell SHP-2 negatively regulates neutrophil adhesion and promotes transmigration by enhancing ICAM-1-VE-cadherin interaction. FASEB J..

[19] Ishida Y (2012). Pivotal role of the CCL5/CCR5 interaction for recruitment of endothelial progenitor cells in mouse wound healing. J Clin Invest..

[20] Xiao Q (2012). TNF-alpha increases bone marrow mesenchymal stem cell migration to ischemic tissues. Cell Biochem Biophys..

[21] Segers VF (2006). Mesenchymal stem cell adhesion to cardiac microvascular endothelium: activators and mechanisms. Am J Physiol Heart Circ Physiol..

[22] De Becker A (2007). Migration of culture-expanded human mesenchymal stem cells through bone marrow endothelium is regulated by matrix metalloproteinase-2 and tissue inhibitor of metalloproteinase-3. Haematologica..

[23] Ries C (2007). MMP-2, MT1-MMP, and TIMP-2 are essential for the invasive capacity of human mesenchymal stem cells: differential regulation by inflammatory cytokines. Blood..

[24] Petit I (2002). G-CSF induces stem cell mobilization by decreasing bone marrow SDF-1 and up-regulating CXCR4. Nat Immunol..

[25] Liu AR (2013). Activation of canonical wnt pathway promotes differentiation of mouse bone marrow-derived MSCs into type II alveolar epithelial cells, confers resistance to oxidative stress, and promotes their migration to injured lung tissue *in vitro*. J Cell Physiol..

[26] Shen Q (2015). Paracrine factors from mesenchymal stem cells attenuate epithelial injury and lung fibrosis. Mol Med Rep..

[27] Zhao F (2019). Pretreatment with G-CSF Could Enhance the Antifibrotic Effect of BM-MSCs on Pulmonary Fibrosis. Stem Cells Int..

[28] Adachi K (2003). Effects of granulocyte colony-stimulating factor (G-CSF) on bleomycin-induced lung injury of varying severity. Toxicol Pathol..

[29] Zhang F (2011). Mobilization of bone marrow cells by CSF3 protects mice from bleomycin-induced lung injury. Respiration..

[30] Malli F (2013). Endothelial progenitor cells in the pathogenesis of idiopathic pulmonary fibrosis: an evolving concept. PLoS One..

[31] Fadini GP, Schiavon M, Rea F, Avogaro A, Agostini C (2007). Depletion of endothelial progenitor cells may link pulmonary fibrosis and pulmonary hypertension. Am J Respir Crit Care Med..

[32] Nakashima T (2013). Lung bone marrow-derived hematopoietic progenitor cells enhance pulmonary fibrosis. Am J Respir Crit Care Med..

[33] Ashcroft T, Simpson JM, Timbrell V (1988). Simple method of estimating severity of pulmonary fibrosis on a numerical scale. J Clin Pathol..

