# Supplementary material for: G-CSF Inhibits Pulmonary Fibrosis by Promoting BMSC Homing to the Lungs via SDF-1/CXCR4 Chemotaxis
Source: Sci Rep. 2020 Jun 29;10:10515. doi: 10.1038/s41598-020-65580-2 (PMC7324625; doi:10.1038/s41598-020-65580-2)
Supplement: Supplementary file 1 — Supplementary Information. [file 41598_2020_65580_MOESM1_ESM.pdf]

G-CSF Inhibits Pulmonary Fibrosis by Promoting BMSC Homing to the Lungs via SDF-1/CXCR4 Chemotaxis

Fei-yan ZHAO[1,2], Tian-yin CHENG[2], Lei YANG[2], Yan-hong HUANG[1], Chen LI[4], Jian-zhong HAN[1], Xiao-hong LI[1], Li-juan FANG[1], Dan-dan FENG[1], Yi-ting TANG[1], Shao-jie YUE[5], Si-yuan TANG[5], Wei LIU[3,*], Zi-qiang LUO[1,*1]

[1]Department of Physiology, Xiangya School of Medicine, Central South University, Changsha, Hunan 410008, China; [2]College of Veterinary Medicine, Hunan Agricultural University, Changsha, Hunan 410128, China; [3]Xiangya Nursing School, Central South University, Changsha, Hunan 410013, China; [4]Department of Physiology, Changzhi Medical College, Changzhi, Shanxi 046000, China; [5]Department of Pediatrics, Xiangya Hospital, Central South University, Changsha, Hunan 410008, China.

Supplementary Figure 1. The protective effects of preventative G-CSF treatment on BLM-induced pulmonary fibrosis. The mice were administrated with G-CSF (60 μg/kg/d) subcutaneously from Day 1 to Day 3, and the lung tissues were harvested on Day 21 for further testing. (A) The pathological examination of the lung sections utilized H&E staining (*upper row*) and Masson's trichrome staining (*lower row*) (scale bar =100 μm, ×100). (B) The Ashcroft scoring method was used to determine the severity of pulmonary fibrosis. (C) The collagen content was quantified by HYP assay. (D) The mRNA expression levels of collagen I and III were also determined by real-time PCR. n=3-6. *P<0.05 between the indicated groups. Bars: mean ± SD.

[1]*Correspondence: luoziqiang@csu.edu.cn; liuw079@csu.edu.cn
zfy277178374@163.com (F.Z.); hn5368@163.com (T.C.); 277670218@qq.com (L.Y.); yanhong0806@163.com (Y.H.); Chen.physiology@outlook.com (C.L.); 765183986@qq.com (J.H.); lixiaohong01@126.com (X.L.);

1341711956@qq.com (L.F.); fengdandanph@163.com (D.F.); 109356513@qq.com (Y.T.); shaojieyue@163.com (S.Y.); siyuantang1966@126.com (S.T.).

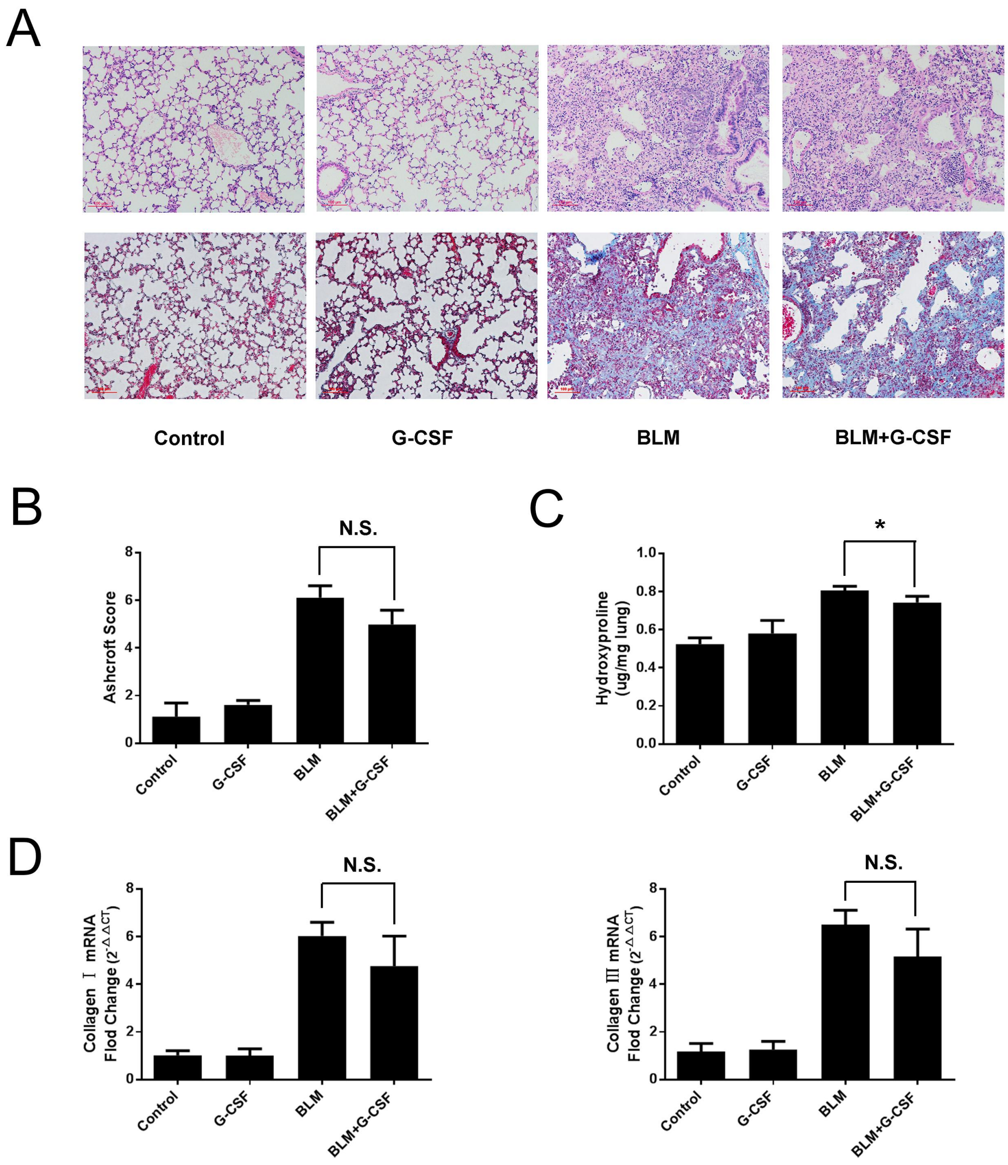
A
Control
G-CSF
BLM
BLM+G-CSF
B
N.S.
Ashcroft Score
0
2
4
6
8
Control
G-CSF
BLM
BLM+G-CSF
C
*
Hydroxyproline (ug/mg lung)
0.0
0.2
0.4
0.6
0.8
1.0
Control
G-CSF
BLM
BLM+G-CSF
D
N.S.
Collagen Ⅰ mRNA Flod Change ($2^{-\Delta\Delta CT}$)
0
2
4
6
8
Control
G-CSF
BLM
BLM+G-CSF
N.S.
Collagen Ⅲ mRNA Flod Change ($2^{-\Delta\Delta CT}$)
0
2
4
6
8
Control
G-CSF
BLM
BLM+G-CSF
